# Long non-coding RNA MIMT1 promotes retinoblastoma proliferation via sponging miR-153-5p to upregulate FGF2

**DOI:** 10.1016/j.heliyon.2024.e34243

**Published:** 2024-07-06

**Authors:** Bin Wang, Ruyuan Cai, Tingting Sun, Zhufang Yang, Hongjie Zhang

**Affiliations:** Department of Ophthalmology, Yancheng Third People's Hospital, Affiliated Hospital 6 of Nantong University, 75 Juchang Street, Yancheng, 224005, China

**Keywords:** lncRNAs MIMT1, miR-153-5p, FGF2, Retinoblastoma

## Abstract

With the rapid development of biotechnology, long non-coding RNAs (lncRNAs) have shown promising potential for cancer treatment and may become novel therapeutic targets. This study aimed to explore the roles of lncRNAs in retinoblastoma (RB). It involves analysing differentially expressed lncRNAs in RB and normal tissues from the GSE111168 and GSE125903 datasets, further validating them in RB cells. Our findings determined that lncRNA MIMT1 was upregulated in RB cell lines and tissues. In WERI-Rb1 and Y79 cells, silencing MIMT1 significantly inhibited cell proliferation, whereas MIMT1 overexpression enhanced cell proliferation. Furthermore, *in vivo* xenograft experiments demonstrated that MIMT1 knockdown suppressed tumour volume and weight. Subsequent mechanistic investigations showed that MIMT1 upregulates fibroblast expression of FGF2 by binding to miR-153-5p, ultimately promoting RB cell proliferation. This suggest that MIMT1 functions as an oncogene in RB and potentially serves as a molecular marker for diagnostic and prognostic assessments. Thus, the MIMT1/miR-153-5p/FGF2 pathway is a promising therapeutic target for RB treatment.

## 1 Background

Retinoblastoma (RB) is globally recognised as the predominant primary intraocular malignant tumour in children, accounting for approximately 6 % of all paediatric malignant tumours [1]. It primarily manifests in the retinal nuclear layer, and is prone to intracranial and distant metastases, leading to significant visual damage and endangering the lives of children. RB treatments continue to evolve and include eye removal, local treatment of ocular tumours (cryotherapy, photocoagulation, and transpupillary warming), and radiotherapy. However, despite these advancements, the overall survival rate of patients with RB has not improved significantly, and its pathogenesis remains unclear [2]. Advancements in modern cellular and molecular biology have illustrated that the onset of RB is primarily associated with abnormalities in molecular and signalling pathways. Gene therapy has become the focus of RB research because of its targeted and reduced adverse effects [3].

Long non-coding RNA (lncRNAs) are non-coding RNA molecules with transcripts that extend beyond 200 nt and display limited or no capability in encoding proteins [4]. lncRNAs are crucial for a wide range of physiological processes, including apoptosis, proliferation, differentiation, chromatin modification, cell cycle regulation, and gene recombination editing [5]. Numerous experimental studies have identified abnormal lncRNA expression in various tumours. These dysregulated lncRNAs are closely correlated with the pathological processes of tumour occurrence, development, metastatic infiltration, and recurrence. This association suggests that lncRNAs either promote or inhibit cancer, potentially serving as new classes of specific tumour biomarkers [[6], [7], [8]]. This offers new prospects for tumour diagnosis and treatment. Currently, reports indicate that the dysregulated expression of various lncRNAs in RB is closely associated with RB development [[9], [10], [11], [12]]. However, research on lncRNAs in RB is relatively limited compared to that in other malignancies.

This study focused on analysing the differentially expressed lncRNAs between RB and retinal tissues using the Gene Expression Omnibus (GEO) database (GSE111168 and GSE125903). Specifically, lncRNAs exhibiting simultaneous differential expression in the two databases, such as MER1 repeats containing imprinted transcript 1 (MIMT1), were selected for further examination. These findings revealed high MIMT1 expression in RB tissues and cells, highlighting its pivotal role in RB development. Considering the absence of prior reports on MIMT1 in RB, this study focused on evaluating the impact of MIMT1 on RB cells and elucidating its underlying mechanisms.

## 2 Methodology

### 2.1 Expression profiling data and correlation analysis

Expression profiling data of lncRNAs exhibiting differential expression between RB and healthy retinal tissues were acquired from the GEO databases, specifically GSE111168 (https://www.ncbi.nlm.nih.gov/geo/query/acc.cgi?acc=GSE111168) and GSE125903 (https://www.ncbi.nlm.nih.gov/geo/query/acc.cgi?acc=GSE125903). An unpaired *t*-test was utilized to identify the differentially expressed lncRNAs between RB and healthy retinal tissues. Differentially expressed lncRNAs with fold change ≥4 and adj *P*-value <0.001 were used for the subsequent analysis. Spearman correlation was utilized to evaluate MIMT1 and FGF2 expression levels using data from GSE110811 (https://www.ncbi.nlm.nih.gov/geo/query/acc.cgi?acc=GSE110811).

### 2.2 Cell culture and transfection

RB cells (WERI-RB1 (HTB-169) and Y79 (HTB-18)) and human retinal epithelial cells (ARPE-19 (CRL-2302)) were procured from the American Type Culture Collection (ATCC). These cells were treated with RPMI-1640 medium (Gibco, USA) enriched with 10 % foetal bovine serum, 100 U/mL penicillin, and 0.1 mg/mL streptomycin under 5 % CO_2_ at 37 °C.

To achieve MIMT1 or FGF2 overexpression, the full-length MIMT1 sequence (NR_024059.2) or FGF2 sequence (NM_001361665.2) was synthesized, and inserted into the pLVX-Puro Plasmid to construct MIMT1 or FGF2 overexpression vectors (Shanghai, China). The online siRNA selection program tool (https://sirna.wi.mit.edu) was used to design the shRNA sequence targeting MIMT1 and FGF2 coding sequence. The MIMT1 short hairpin RNAs (shRNA) sequences (5′-GGATCCCCCACTGAGATGCTCAAATTTCAAGAGAATTTGAGCATCTCAGTGGGTTTTTT-3′) or FGF2 shRNA sequence (5′-GGATCCCTGGGCAGAAAGCTATACTTTCAAGAGAAGTATAGCTTTCTGCCCAGTTTTTT-3′) was synthesized, and inserted into the pLKO.1-Puro Plasmid to construct MIMT1 or FGF2 down-regulated vectors (Shanghai, China). Additionally, miR-153-5p inhibitors, miR-153-5p mimics, and negative controls were obtained from Yansai Biotechnology. The sense and antisense sequences of miR-153-5p mimics and miR-153-5p inhibitors, including the negative controls were shown in Supplementary Table 1. Cell transfection was performed using Lipofectamine 2000 (Invitrogen, USA) following the manufacturer's protocol. Further experiments were performed 24 h after transfection.

### 2.3 Quantitative real-time polymerase chain reaction (qRT-PCR)

Total RNA was extracted using the TRIzol reagent (Invitrogen, USA). PrimeScript RT Reagent Kit (TaKaRa, Japan) was used to synthesis lncRNA and mRNA cDNA, and Mir-X™ miRNA First Strand Synthesis Kit (TaKaRa, Japan) was used to synthesis miRNA cDNA. qRT-PCR was performed using either SYBR PrimeScriptTM miRNA RT-PCT or SYBR Premix Ex *Taq*II Kit (TaKaRa, Japan) to assess the relative expression levels of mRNA, lncRNA, or miRNA. Moreover, GAPDH served as an internal reference to identify mRNA and lncRNAs, and U6 was used for miRNA detection. The primers used are listed in Supplementary Table 2.

### 2.4 Western blot analysis

Radioimmunoprecipitation assay lysis buffer was used to extract total proteins, and their concentrations were determined using a BCA kit (Sigma-Aldrich, USA). Equivalent amounts of protein were separated by SDS-PAGE and transferred onto a PVDF membrane (Millipore, USA). Additionally, 5 % skimmed milk powder was used to block the membrane for 1 h. This was followed by overnight exposure to primary antibodies at 4 °C, and subsequent room temperature exposure to secondary antibodies for 1 h. The protein bands were visualised using ChemiDoc (Bio-Rad, USA). The antibodies employed included: anti-FGF2 (1:1000 dilution; ab92337, Abcam, USA) and anti-β-Actin (1:10000 dilution; ab8226, Abcam, USA).

### 2.5 Nucleoplasmic separation

Nuclear and cytoplasmic extraction reagents (Thermo Scientific, USA) were used to separate the cytoplasmic and nuclear components of WERI-RB1 and Y79 cells. Subsequently, RNA was extracted for qRT-PCR analysis, with GAPDH and U6 serving as cytoplasmic and nuclear markers, respectively.

### 2.6 Cell count Kit-8 (CCK-8) assay for cell proliferation detection

A 100 μL suspension of transfected cells (approximately 1 × 10^3^ cells) was inoculated into a 96-well plate and cultured. At each monitoring time point (24, 48, 72, and 96 h), CCK-8 solution (10 μL) (Dojindo Laboratories, Japan) was added into the wells. The plate was gently tapped to ensure thorough mixing and prevent the formation of bubbles that could affect the optical density (OD). Following a 2-h culture at 37 °C, the OD value at 450 nm was computed using Bio-Tek EPOCH2 (BioTek, USA). This experimental procedure was repeated in triplicate to ensure precision and reliability.

### 2.7 Clone formation experiment

The transfected cells were resuspended and then seeded into a culture dish with 10 mL of the pre-warmed culture medium (37 °C). Subsequently, the dish was incubated in 37 °C with 5 % CO_2_ for 14 d. The cells were then washed two times with phosphate-buffered saline and fixed in 4 % paraformaldehyde for 20 min. Subsequently, the cells were exposed to 0.1 % crystal violet at room temperature for 30 min. Then, cell clones with a diameter surpassing 0.1 mm were counted under a microscope.

### 2.8 Selection of miRNA adsorbed by lncRNA

Firstly, search for the sequence of lncRNA through the National Center for Biotechnology Information (NCBI). Secondly, predict potential miRNAs adsorbed by the lncRNA using the miRDB database (https://mirdb.org/), select the top 5 target scores for validation. Finally, validate the selected miRNAs in cells through qRT-PCR, Western blot, and Dual-luciferase Reporter Assay.

### 2.9 Dual-luciferase reporter assay

A database (https://mirdb.org/) was used to predict the binding sites of MIMT1, FGF2, and miR-153-5p. Subsequently, MIMT1 sequences containing the predicted miR-153-5p binding site (AAAAATGA) or a mutated sequence (TTTTTACT) were chemically synthesized and purchased from Yansai Biotechnology (Shanghai, China), and inserted into the psiCHECK-2 luciferase reporter vector (Shanghai, China). FGF2 sequences containing the predicted miR-153-5p binding site (AAAAATG) or a mutated sequence (TTTTTAC) were chemically synthesized and purchased from Yansai Biotechnology (Shanghai, China) These plasmids were then co-transfected with miR-153-5p mimics of Y79 and WERI-RB1 cells. After 48 h, luciferase activity was evaluated using the dual-luciferase reporter assay system (Promega, USA) following the manufacturer's protocols.

### 2.10 Animal experiments

WERI-Rb1 cells (5 × 10^6^) were suspended in 150 μL of FBS-free media and subcutaneously administered into the backs of BALB/c nude mice (4-weeks-old) after sh-MIMT1 or sh-normal control (sh-NC) transfection. Each experimental group comprised six mice. The status of the nude mice was systematically observed and recorded every 3 d to monitor tumour growth and volume. Tumour volume was recorded weekly and computed using the following formula: tumour volume = (square of width × length)/2. Thirty days after cell implantation, the mice were anaesthetised with 1 % sodium pentobarbital (50 mg/kg; Sigma, USA) and decapitated. The excised tumours were weighed. The animal experiments were conducted in accordance with the principles and protocols prescribed in the Guide for the Care and Use of Animals at the Ethics Committee of Jiangsu Medical Vocational College (SYXK-2023-0005).

### 2.11 Statistics

GraphPad Prism 9.0 statistical software (Dotmatics, England) was utilized for statistical analyses. The differences in each index between the two groups were assessed via an independent sample *t*-test. Overall variance across multiple groups was investigated through one way analysis of variance, and two-by-two comparisons were executed via least significant difference *t*-test. Statistical significance was set as *P* < 0.05.

## 3 Results

### 3.1 Elevated expression of lncRNAs MIMT1 in RB

**Fig. 1:**
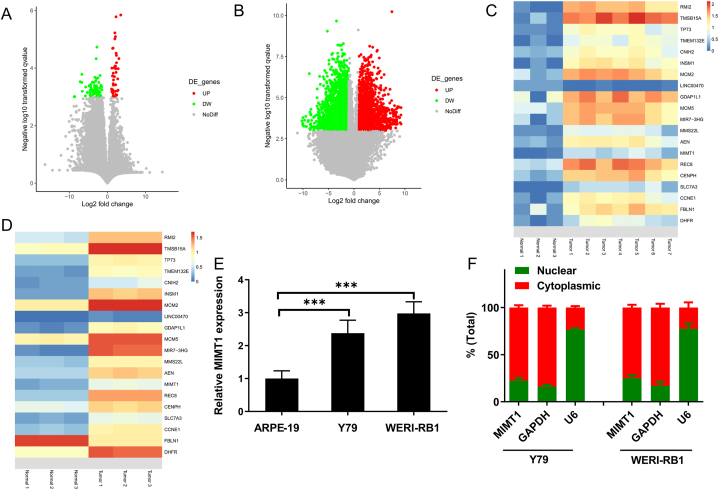
(A) Volcano map illustrating differential gene expression in GSE125903 (fold change >4.0 and *P* < 0.001). (B) Volcano plot depicting differential gene expression in GSE111168 (fold change >4.0 and *P* < 0.001). (C) Heatmap displaying representative differentially expressed genes from seven RB and three healthy retinal tissues in the GSE125903 dataset. (D) Heatmap illustrating representative differentially expressed genes from three RB and three healthy retinal tissues in the GSE111168 dataset. (E) qRT-PCR outcomes of MIMT1 expression in retinal epithelial (ARPE-19) and RB cells (Y79 and WERI-RB1) (F) Nucleoplasmic separation assay depicting the distribution of MIMT1 in cells. (***P < 0.001, **P < 0.01, *P < 0.05).

We compared RB with healthy retinas in the exome sequencing datasets GSE111168 and GSE125903, numerous mRNAs exhibited simultaneous differential expression in both datasets (Fig. 1A–D). Fig. 1C and D respectively display the representative differentially expressed genes between RB and normal retinal tissues in the GSE125903 and GSE111168 datasets. However, among the lncRNAs, only MIMT1, MIR7-3HG, and LINC00470 met the fold change ≥4 criterium with a significance level of *P* < 0.001. MIR7-3HG's association with RB has been demonstrated previously [13]. Additionally, LINC00470 exhibited elevated expression in RB according to GSE111168 and decreased expression in RB according to GSE125903. MIMT1 is shown to be upregulated in RB compared to normal retinal tissues in both the GSE125903 and GSE111168 datasets. Therefore, MIMT1 was selected for subsequent studies. Subsequently, MIMT1 expression was assessed using qRT-PCR in RB (WERI-RB1 and Y79) and retinal epithelial cells (ARPE-19), indicating a substantial elevation in MIMT1 expression in RB cells compared to MIMT1 expression in retinal epithelial cells (Fig. 1E). A nuclear-cytoplasmic fractionation assay revealed that MIMT1 was primarily localised in the cytoplasm (Fig. 1F).

### 3.2 Proliferative impact of MIMT1 on RB cells *In vivo* and *In vitro*

**Fig. 2:**
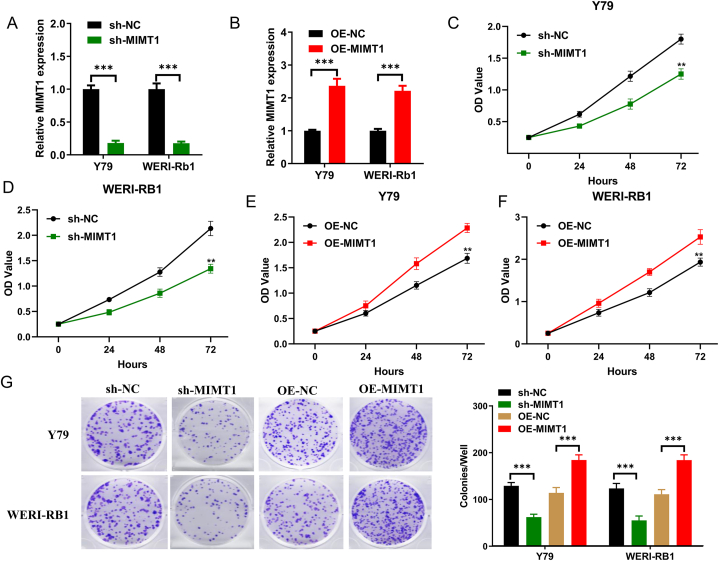
Using qRT-PC to validate the impact of MIMT1 expression modulation in Y79 and WERI-RB1 cell lines. (A) Downregulated and (B) upregulated MIMT1. After downregulating the MIMT1 expression, CCK-8 assayed the proliferative capacity of (C) Y79 and (D) WERI-RB1 cells. After MIMT1 upregulation, CCK-8 detected the proliferative ability of (E) Y79 and (F) WERI-RB1 cells. After upregulation or downregulation of MIMT1 expression, a clone formation assay analysed the proliferative capacity of Y79 and WERI-RB1 cells (G). (***P < 0.001, **P < 0.01, *P < 0.05). sh-: short hairpin; OE-: overexpression.

**Fig. 3:**
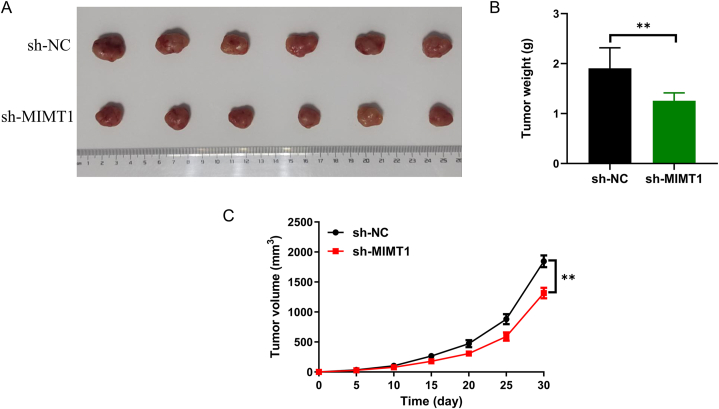
(A) Tumours acquired from the control and MIMT1 shRNA-treated groups were treated for 30 d *in vivo.* (B) Tumour weights of the control and MIMT1 shRNA-treated groups. (C) Tumour growth curves of the control and MIMT1 shRNA-treated groups. (***P < 0.001, **P < 0.01, *P < 0.05). sh-: short hairpin.

To evaluate the influence of MIMT1 on RB cell proliferation, MIMT1 expression was modulated in RB cells (Y79 and WERI-RB1) by lentiviral transfection with an overexpression plasmid or shRNA. Fig. 2A and B shows the post-transfection expression of MIMT1. To assess the proliferative capacity of RB cells, we conducted CCK-8 and clone formation assays. The CCK-8 assay demonstrated that a reduction in MIMT1 expression considerably impeded the proliferative viability of RB cells (Fig. 2C–D). Using clone formation assays, MIMT1 upregulation increased the proliferative viability and clone formation in RB cells (Fig. 2E–F). While, MIMT1 downregulation suppressed cell clone formation (Fig. 2G). Subsequently, we assessed MIMT1 involvement in tumour growth *in vivo* by injecting stably downregulated MIMT1 (achieved using MIMT1 shRNA) into the subcutaneous tissues of the nude mice cell lines, Y79 and control. The mice were then raised for 30 d (Fig. 3A). Our findings suggest that the MIMT1 shRNA group exhibited notably decreased tumour weight and volume compared to the control group (Fig. 3B and C). In conclusion, MIMT1 knockdown significantly inhibits RB cell growth *in vitro* and *in vivo*.

### 3.3 MIMT1 regulates the proliferative capacity of RBs by inhibiting mir-153-5p

**Fig. 4:**
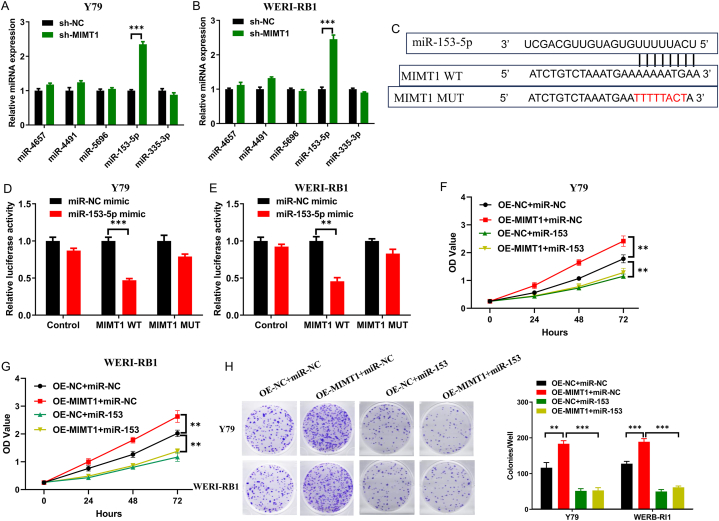
(A–B) Changes in candidate miRNA expression assessed via qRT-PCR after MIMT1 downregulation in Y79 and WERI-RB1 cells. (C) The binding sequences of MiR-153-5p and MIMT1 as well as the mutated sequences of MIMT1. (D–E) Verification of direct binding between MIMT1 and miR-153-5p via luciferase reporter gene assay in WERI-RB1 and Y79 cells. (F–G) CCK-8 assay to test the proliferative capacity of WERI-RB1 and Y79 cells transfected with OE-NC + miR-NC, OE-MIMT1+miR-NC, OE-NC + miR-153-5p mimic, and OE-MIMT1+miR-153-5p mimic. (H) Clone formation assays were performed to detect the proliferative capacity of Y79 and WERI-RB1 cells transfected with OE-NC + miR-NC, OE-MIMT1+miR-NC, OE-NC + miR-153-5p mimic, and OE-MIMT1+miR-153-5p mimic. (***P < 0.001, **P < 0.01, *P < 0.05). sh-: short hairpin; OE-: overexpression.

lncRNAs have the potential to regulate gene expression. Those highly expressed in the cytoplasm can function as competing endogenous RNAs (ceRNAs) and engage in competitive binding with miRNAs. This mechanism regulates the downstream target genes of miRNAs, ultimately inhibiting their biological functions [14]. The cytoplasmic localisation of MIMT1 was verified using a nucleoplasmic assay (Fig. 1F), and the acquired data suggested its potential involvement as a ceRNA. The miRDB database (https://mirdb.org/) predicted a higher likelihood of miR-4657, miR-4491, miR-5696, miR-153-5p, and miR-335-3p binding to MIMT1. Moreover, MIMT1 expression in WERI-RB1 and Y79 cell lines was reduced to evaluate the levels of the predicted miRNAs using qRT-PCR. Our results indicated a notable upregulation in miR-153-5p (Fig. 4A–B). The constructed MIMT1 WT and MUT sequences were subsequently inserted into the luciferase reporter plasmid, psiCheck2 (Fig. 4C), demonstrating a notable decrease in luciferase activity in the Y79 and WERI-RB1 cell lines following the co-transfection of MIMT1 WT with the miR-153-5p mimic. However, this mechanism did not induce a significant alteration in luciferase activity (Fig. 4D–E). The acquired data indicated that MIMT1 can bind to miR-153-5p directly using the mutated site.

A rescue assay was conducted to investigate whether MIMT1 regulated the proliferative capacity of RB cells by suppressing miR-153-5p expression. CCK-8 and clone formation assays revealed that, compared to solely upregulating MIMT1, co-transfecting the WERI-RB1 and Y79 cells with the OE-MIMT1 and miR-153-5p mimic, respectively, resulted in the re-suppression of elevated MIMT1 expression and cell proliferation (Fig. 4F–H). Therefore, MIMT1 regulates the proliferative ability of RB cells by inhibiting miR-153-5p.

### 3.4 MIMT1 upregulates FGF2 expression via competitive binding to mir-153-5p, thereby upregulating RB proliferative capacity

**Fig. 5:**
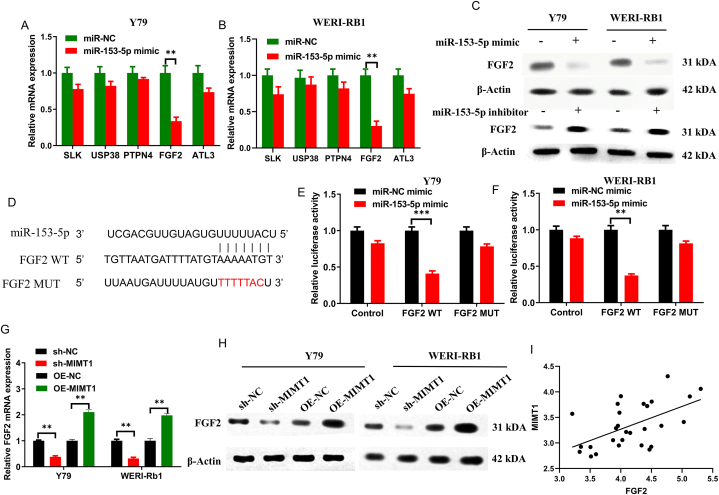
(A–B) Changes in candidate mRNAs expression detected via qRT-PCR following miR-153-5p overexpression in Y79 and WERI-RB1 cells. (C) Detecting the FGF2 protein in Y79 and WERI-RB1 cells overexpressing miR-153-5p using western blotting. (D) MiR-153-5p and FGF2 binding sequences and FGF2 mutation sequences. (E–F) Using the luciferase reporter gene assay the direct binding between FGF2 and miR-153-5p in WERI-RB1 and Y79 cells was verified. (G) Using qRT-PCR to detect the mRNA of FGF2 in WERI-RB1 and Y79 cells that contained upregulated or downregulated MIMT1. (H) Western blotting detected the FGF2 protein in WERI-RB1 and Y79 cells that contained upregulated or downregulated MIMT1. (I) The association between MIMT1 and FGF2 in 28 RB tissues. (***P < 0.001, **P < 0.01, *P < 0.05). sh-: short hairpin; OE-: overexpression.

The miRDB (http://mirdb.org/) and TargetScan (http://www.targetscan.org/) databases, along with related literature, identified several potential target genes of miR-153-5p. These candidate genes included *SLK, USP38, PTPN4, ATL3*, and *FGF2*. miR-153-5p expression was upregulated in the Y79 and WERI-RB1 cell lines, and qRT-PCR results showed notable alterations in the mRNA levels of *FGF2* (Fig. 5A–B). Subsequently, western blotting was performed to evaluate FGF2 protein expression in the WERI-RB1 and Y79 cells. Our findings showed that miR-153-5p overexpression reduced FGF2 protein expression, and miR-153-5p down-expression increased FGF2 protein expression (Fig. 5C). The FGF2 WT and MUT sequences were constructed and inserted into the luciferase reporter plasmid psiCheck2 (Fig. 5D). Dual-luciferase reporter assays were then performed. The results illustrated that increased miR-153-5p expression considerably lowered the luciferase activity of the FGF2-WT vector. In contrast, there was no notable impact on the empty or FGF2-MUT vectors. (Fig. 5E–F), confirming that FGF2 is a direct target gene of miR-153-5p.

MIMT1 alteration impact on FGF2 expression was validated by regulating MIMT1 expression in WERI-RB1 and Y79 cell lines. qRT-PCR and western blotting assays demonstrated that MIMT1 upregulation elevated FGF2 mRNA and its protein levels, whereas MIMT1 downregulation reduced FGF2 mRNA and its protein levels (Fig. 5G–H). Additionally, analysis of the GSE110811 dataset revealed that MIMT1 was positively correlated with FGF2 expression in 28 RB tissue samples (R = 0.55, *P* = 0.002) (Fig. 5I).

**Fig. 6:**
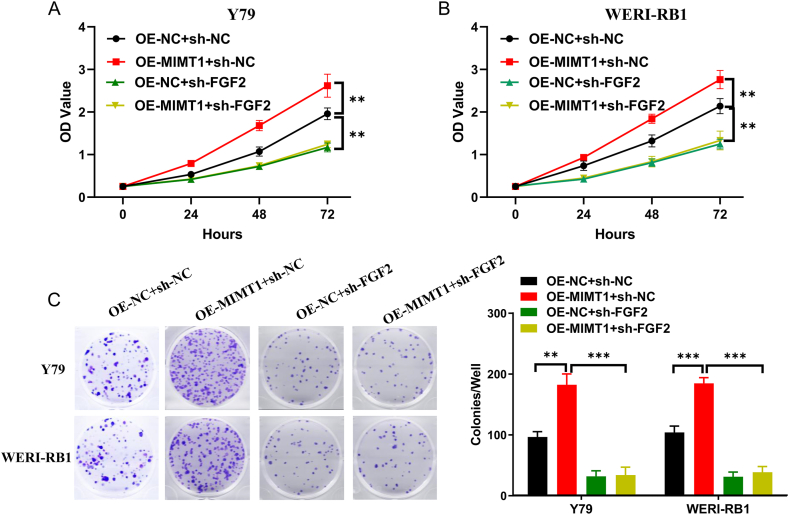
Assessment of proliferative capacity in Y79 and WERI-RB1 cells transfected with OE-NC + sh-NC, OE-MIMT1+sh-NC, OE-NC + sh-FGF2, and OE-MIMT1+ sh-FGF2 (A–B) CCK-8 assay. (C) Clone formation assay. (***P < 0.001, **P < 0.01, *P < 0.05). sh-: short hairpin; OE-: overexpression.

Rescue experiments, clone formation and CCK-8 assays, were conducted to ascertain whether MIMT1 promotes tumour proliferation through FGF2. Y79 and WERI-RB1 cells were transfected with four different treatments: (1) OE-NC + sh-NC, (2) OE-MIMT1+sh-NC, (3) OE-NC + sh-FGF2, or (4) OE-MIMT1+ sh-FGF2. Our findings demonstrated that MIMT1 overexpression did not enhance the proliferative ability of WERI-RB1 and Y79 cells when FGF2 was silenced (Fig. 6A–C). In conclusion, MIMT1 promoted RB cell proliferation by competitively binding to miR-153-5p and upregulating FGF2 expression.

## 4 Discussion

RB is the most prevalent paediatric intraocular primary malignant tumour, with a global incidence of 1 in 14,000–20,000 newborns [1]. The absence of early diagnostic techniques and efficacious treatments significantly contributes to a less favourable prognosis for most paediatric RB cases. This challenge is notably pronounced in developing countries, where limited resources and healthcare infrastructure exacerbates the difficulties faced in RB management. Therefore, the identification of precise and effective biomarkers or therapeutic targets is promising for advancing RB diagnosis and treatment. Recently, the focus on lncRNAs has intensified in tumour research, garnering increasing attention for their biological roles in RB development. Ni et al. observed a reduction in PI3K/Akt signalling activity via PI3Kγ silencing, by lncRNA CANT1, inhibiting RB tumourigenesis [9]. Similarly, Wu et al. reported that inhibiting lncRNA PVT1 markedly suppressed various aspects of RB cell characteristics, including proliferation, invasion, migration, and cell cycle progression, triggering apoptosis [7]. lncRNA MBNL1-AS1 deactivated the Wnt/β-catenin signalling pathway in RB by targeting miR-338-5p [15]. Additionally, lnc00152 induces EMT via the miR-30d/SOX9/ZEB2 pathway, consequently promoting RB cell invasion and metastasis [16]. However, the precise roles of lncRNAs in RB progression remains unclear. Herein, lncRNA MIMT1, which is closely associated with RB, was identified by analysing the GSE111168 and GSE125903 datasets. Subsequent functional assays substantiated that MIMT1 upregulation enhanced the proliferative capacity of RB cells, whereas MIMT1 downregulation impeded the proliferative ability of RB cells. Animal experiments further validated that MIMT1 knockdown inhibits tumour growth. Although we are eager to examine the clinical significance of MIMT1, our hospital does not have a sufficient number of RB for analysis.

lncRNAs interacts with miRNAs through complementary sequences, competitively attenuating the binding between miRNAs and target mRNA. This process serves as a pivotal regulatory mechanism for gene expression at the post-transcription stage [17]. Herein, candidate miRNAs for MIMT1 were assessed using bioinformatics analysis. A dual-luciferase reporter assay confirmed the presence of a binding between MIMT1 and miR-153-5p. Moreover, miR-153-5p is involved in the onset and progression of numerous malignant tumours [[18], [19], [20]]. A previous study, indicated that miR-153-5p overexpression increases drug sensitivity to paclitaxel in breast cancer cells. This enhancement effectively suppressed the proliferative capacity and migratory potential of breast cancer cells while promoting apoptosis [21]. In addition, miR-153-5p expression is considerably suppressed in ovarian cancer compared to miR-153-5p expression in paraneoplastic tissues. This substantial reduction decreased the proliferative and invasive capacities of ovarian cancer cells, exerting a tumour-suppressive effect [22]. Additionally, Xie et al. demonstrated that miR-153-5p overexpression suppressed the invasive and migratory capacity of glioblastoma [18]. Chen et al. demonstrated that reducing miR-153-5p expression in hepatocellular carcinoma cells induces the upregulation of ARHGAP18, subsequently enhancing their migration [23]. These findings confirm the tumour suppressive role of miR-153-5p. However, contradictory results have been reported in other studies. Li et al. observed notable miR-153-5p upregulation in renal clear-cell carcinoma, promoting cancer development [20]. In addition, this study demonstrated that FGF2 is a direct target gene of miR-153-5p and that miR-153-5p modulates the proliferative ability of RB cells by inhibiting FGF2 expression. FGFs actively participate in biological development, tissue homeostasis maintenance, and catalyse processes such as angiogenesis and tumour progression [24]. FGF2, a well-studied member of the FGF family, exhibits diverse biological functions in various cellular and organ systems [25]. Elevated expression of FGF2 characterises numerous human tumours, emphasising its crucial contribution in the proliferation and invasion of cancer cells [26]. In prostate cancer, FGF2 is substantially upregulated and has a notable influence on the malignant progression of this condition. FGF2 knockdown in mice notably delayed the onset and progression of prostate cancer [27]. In breast cancer cells, FGF2 exhibits high expression, acting as an anti-apoptotic agent, and induces invasion [28]. FGF2 knockdown in non-small cell lung cancer impedes cell proliferation, colony formation, migration, and invasion [29]. In renal cancer, FGF2 enhances cell proliferation by triggering the FGFR2 signalling pathway [30]. Additionally, FGF2 suppression markedly impedes the proliferative capacity and invasiveness of cervical cancer cells [31]. The present study confirms that MIMT1 positively regulates FGF2 expression in RB cells. Furthermore, FGF2 overexpression reversed the MIMT1 downregulation-induced reduced proliferative capacity of RB cells. Therefore, MIMT1 enhances the proliferative capacity of RB by modulating the miR-153-5p/FGF2 axis. Thus, the MIMT1/miR-153-5p/FGF2 pathway is a potential therapeutic target for RB. Although the MIMT1/miR-153-5p/FGF2 pathway has been proposed as a potential therapeutic target, the clinical efficacy and safety of these targets should be verified by further studies.

## Ethics approval and consent to participate

The animal experiments were conducted following protocols approved by the Ethics Committee of Jiangsu Medical Vocational College (SYXK-2023-0005) and complied with the national guidelines for the care and use of animals.

## Consent for publication

Not applicable.

## Availability of data and material

The authors confirm that the data supporting the findings of the present study are available in the article, supplementary material and upon reasonable request.

## Funding

None.

## CRediT authorship contribution statement

**Bin Wang:** Writing – original draft, Data curation, Conceptualization. **Ruyuan Cai:** Writing – original draft, Data curation. **Tingting Sun:** Data curation, Conceptualization. **Zhufang Yang:** Software, Data curation. **Hongjie Zhang:** Writing – review & editing, Resources, Funding acquisition, Conceptualization.

## Declaration of competing interest

The authors declare that they have no known competing financial interests or personal relationships that could have appeared to influence the work reported in this paper.
