# Supplementary material for: Long non-coding RNA MIMT1 promotes retinoblastoma proliferation via sponging miR-153-5p to upregulate FGF2
Source: Heliyon. 2024 Jul 6;10(13):e34243. doi: 10.1016/j.heliyon.2024.e34243 (PMC11292528; doi:10.1016/j.heliyon.2024.e34243)
Supplement: Multimedia component 1 [file mmc1.docx]

Supplementary table 1 The sense and antisense sequences of miR-153-5p mimics and miR-153-5p inhibitors, including the negative controls

| **Name** | **Sense (5’-3’)** | **Anti-sense (5’-3’)** |
| --- | --- | --- |
| miR-NC mimic | UUGUACUACACAAAAGUACUG | GUACUUUUGUGUAGUACAAUU |
| miR-153 mimic | UCAUUUUUGUGAUGUUGCAGCU | CUGCAACAUCACAAAAAUGAUU |
| miR-NC inhibitor | CAGUACUUUUGUGUAGUACAA |  |
| miR-1244 inhibitor | AGCUGCAACAUCACAAAAAU |  |

Supplementary table 2 Primer sequences used for qPCR assays

| Gene | Sequence of the primers |
| --- | --- |
| MIMT1-Forward | 5'- TGTAGAAACGAACTGGCTCCTTTTATGT-3' |
| MIMT1-Reverse | 5'- GTTGAGGTCCCAGGCTCATTGTG-3' |
| FGF2-Forward | 5'-AGAAGAGCGACCCTCACATCA-3' |
| FGF2-Reverse | 5'-CGGTTAGCACACACTCCTTTG-3' |
| SLK-Forward | 5'-ACAAGGCCCAGAATAAAGAGACC-3' |
| SLK-Reverse | 5'-AGTTCAAGCATCACAGCATCTAC-3' |
| USP38-Forward | 5'-CAGTGCGAGGCCATGTTTG-3' |
| USP38-Reverse | 5'-CGGTGGTATCGTGCGTAGG-3' |
| PTPN4-Forward | 5'-GCAGAACCTACAATGTACGAGC-3' |
| PTPN4-Reverse | 5'-ACGACATCCAACAAGACTTGC-3' |
| ATL3-Forward | 5'-ACAGCCAGTCAACTGTGAAAG-3' |
| ATL3-Reverse | 5'-CCAGACGACCGTATTCTGTGA-3' |
| miR-153-5p-Forward | 5'-ACACTCCAGCTGGGTCATTTTTGTGATGTTGC-3' |
| miR-153-5p-Reverse | 5'CTCAACTGGTGTCGTGGA-3' |
| GAPDH-Forward | 5'-GGAGCGAGATCCCTCCAAAAT-3' |
| GAPDH-Reverse | 5'-GGCTGTTGTCATACTTCTCATGG-3' |
| U6-Forward | 5'-CTCGCTTCGGCAGCACA-3' |
| U6- Reverse | 5'-AACGCTTCACGAATTTGCGT-3' |
